# Specific and Sensitive Hydrolysis Probe-Based Real-Time PCR Detection of Epidermal Growth Factor Receptor Variant III in Oral Squamous Cell Carcinoma

**DOI:** 10.1371/journal.pone.0031723

**Published:** 2012-02-16

**Authors:** John B. McIntyre, Pinaki Bose, Alexander C. Klimowicz, Nigel T. Brockton, Stephanie Petrillo, Wayne Matthews, Jay Easaw, Anthony Magliocco, Joseph C. Dort

**Affiliations:** 1 Department of Pathology and Laboratory Medicine, Tom Baker Cancer Centre, University of Calgary, Calgary, Canada; 2 Department of Oncology, University of Calgary, Calgary, Canada; 3 Department of Population Health Research, Alberta Health Services - Cancer Care, Calgary, Canada; 4 Department of Surgery (Division of Otolaryngology-Head and Neck Surgery), University of Calgary, Calgary, Canada; 5 Division of Neurosurgery, Department of Clinical Neurosciences, University of Calgary, Calgary, Canada; National Taiwan University Hospital, Taiwan

## Abstract

**Background:**

The tumor-specific EGFR deletion mutant, *EGFRvIII*, is characterised by ligand-independent constitutive signalling. Tumors expressing EGFRvIII are resistant to current EGFR-targeted therapy. The frequency of *EGFRvIII* in head and neck squamous cell carcinoma (HNSCC) is disputed and may vary by specific sub-site. The purpose of this study was to measure the occurrence of *EGFRvIII* mutations in a specific HNSCC subsite, oral squamous cell carcinoma (OSCC), using a novel real-time PCR assay.

**Methodology:**

Pre-treatment Formalin Fixed Paraffin Embedded (FFPE) cancer specimens from 50 OSCC patients were evaluated for the presence of *EGFRvIII* using a novel hydrolysis probe-based real-time PCR assay. EGFR protein expression in tumor samples was quantified using fluorescent immunohistochemistry (IHC) and AQUA® technology.

**Principal findings:**

We detected *EGFRvIII* in a single OSCC patient in our cohort (2%). We confirmed the validity of our detection technique in an independent cohort of glioblastoma patients. We also compared the sensitivity and specificity of our novel real-time *EGFRvIII* detection assay to conventional RT-PCR and direct sequencing. Our assay can specifically detect *EGFRvIII* and can discriminate against wild-type EGFR in FFPE tumor samples. AQUAnalysis® revealed that the presence of *EGFRvIII* transcript is associated with very high EGFR protein expression (98^th^ percentile). Contrary to previous reports, only 44% of OSCC over-expressed EGFR in our study.

**Conclusion and Significance:**

Our results suggest that the *EGFRvIII* mutation is rare in OSCC and corroborate previous reports of EGFRvIII expression only in tumors with extreme over-expression of EGFR. We conclude that EGFRvIII-specific therapies may not be ideally suited as first-line treatment in OSCC. Furthermore, highly specific and sensitive methods, such as the real-time RT-PCR assay and AQUAnalysis® described here, will provide accurate assessment of EGFR mutation frequency and EGFR expression, and will facilitate the selection of optimal tailored therapies for OSCC patients.

## Introduction

The discovery of oncogenic mutations in key genes regulating growth and proliferation has enhanced our understanding of the molecular pathology of cancer [1]. Many of these mutations are shared by tumors arising in distinct tissue types and at diverse anatomical locations, thereby emphasizing their vital importance in carcinogenesis. The epidermal growth factor receptor (EGFR) signalling pathway is frequently dysregulated in several cancer types. EGFR is a membrane-spanning glycoprotein consisting of an extracellular ligand-binding domain, a transmembrane domain, and an intracellular tyrosine kinase domain. EGFR mediates a variety of responses including growth, proliferation, angiogenesis and metastasis [2]. On ligand binding, EGFR can activate two major signalling pathways: Ras/MAPK and PI3K/AKT/mTOR pathways (Reviewed in [3], [4]). EGFR is ubiquitously expressed in normal epithelial tissue but is over-expressed in several cancers including lung, glioblastoma, prostrate, breast, colon, ovary and head and neck. Mutations in the *EGFR* gene have also been described in a variety of cancers.

Our study focuses on the most common and well-characterized EGFR mutant, EGFR class III variant (*EGFRvIII*) [5]. *EGFRvIII* is a cancer-specific deletion of exons 2 to 7 (801 bp) that results in a truncated extracellular domain of EGFR [6]. The corresponding deletion of amino acids 30–297 within the extracellular ligand-binding domain, results in a ligand-independent form of EGFR with constitutive tyrosine kinase activity [7], leading to increased cell proliferation and inhibition of apoptosis [7]. *EGFRvIII* expression is frequently associated with amplification and over-expression of wild-type EGFR [8], [9] and EGFRvIII-expressing tumors are more resistant to radiotherapy and chemotherapy [10], [11].

The *EGFRvIII* mutant was first detected in glioblastomas [12], [13], [14] and approximately 30% of glioblastomas express *EGFRvIII* [15]. *EGFRvIII* has been reported in lung squamous cell carcinomas but not in adenocarcinomas [16], [17]. *EGFRvIII* has also been reported in breast cancer and ovarian cancer [5]. In prostrate tumors, *EGFRvIII* expression increases progressively during the transition from pre-malignant prostate lesions to the malignant phenotype [18].

Previous reports suggest that EGFR is over-expressed in ∼90% of head and neck squamous cell carcinoma (HNSCC) [19], [20], [21] and EGFR over-expression has been linked to the presence of EGFRvIII at other cancer sites [8], [9]. Therefore, recent studies have examined the role of EGFRvIII in HNSCC etiology [21], [22], [23]. However, the frequency of EGFRvIII in head and neck cancer is disputed [24]. Two studies have reported the presence of *EGFRvIII* in 42% of HNSCC patients [22], [23] but another group did not detect any *EGFRvIII* in HNSCC patients [21]. One study, specifically in laryngeal carcinoma patients, reported the presence of *EGFRvIII* in ∼15% of tumor samples [25].

The level of EGFR protein expression in HNSCC can vary according to the specific subsite; for instance, carcinomas of the pharynx and oral cavity tend to have higher EGFR expression than those of the larynx [26]. Therefore, the frequency of *EGFRvIII* expression may also differ in distinct HNSCC subsites. Despite the lack of consensus on the frequency of *EGFRvIII* in HNSCC [24], only one study has discussed the frequency of *EGFRvIII* in specific HNSCC subsites which include malignancies of the oral cavity, pharynx and larynx.

Oral squamous cell carcinoma (OSCC) accounts for approximately 30% of all HNSCCs. The 5-year survival rate for OSCC is approximately 50% and has changed little in the last few decades. This failure of conventional therapies supports the use of novel therapeutic strategies in OSCC. Therapeutic agents that target specific abnormalities in HNSCC have been employed successfully in recent years [27]. The reduced toxicity profile of these agents, compared to conventional chemotherapy and radiotherapy regimens, spares the surrounding non-cancer tissue from the deleterious effects of such therapy. Monoclonal antibodies and small molecule tyrosine kinase inhibitors have been developed to target EGFR in diverse tumor types. The most commonly used anti-EGFR therapy is Cetuximab (ImClone LLC). However, Cetuximab binds in the ligand-binding domain that is deleted in the EGFRvIII mutant form, rendering EGFRvIII-positive tumors refractory to Cetuximab treatment [22]. Consequently, the effective targeting of these novel therapies depends on the sensitive and reliable detection of these specific abnormalities to identify patients who are candidates for these agents.

The use of immunohistochemistry to detect EGFRvIII protein expression in clinical samples is restricted by existing patents [28]. Also, the limited availability of frozen tissue poses a significant challenge to the use of conventional reverse transcriptase-PCR (RT-PCR) and Southern blot assays for the detection of *EGFRvIII* since they require high nucleic acid integrity. Thus, robust and sensitive methods for detecting *EGFRvIII* in FFPE samples are required. Several real-time PCR assays have been developed for the detection of *EGFRvIII* in FFPE tumor samples [23], [28]. In the present study, we developed a highly sensitive and specific hydrolysis probe-based real-time PCR (real-time RT-PCR) assay for the detection of *EGFRvIII* in FFPE tumor samples. This technique relies on TaqMan® hydrolysis probe-based chemistry and achieves higher sensitivity than presently employed SYBR green-based techniques. We demonstrated the specificity of our method in *EGFRvIII*-positive and negative cell lines and then validated our method by detecting *EGFRvIII* in a cohort of glioblastoma patients. Finally, we screened a cohort of patients with OSCC to determine the frequency of *EGFRvIII* mutations. Our results suggest that the *EGFRvIII* mutation is rare in OSCC. Using quantitative fluorescence immunohistochemistry and AQUA® technology, we analyzed EGFR protein expression in our OSCC cohort and found that exceptionally high EGFR expression in carcinoma cells was associated with *EGFRvIII* positivity.

## Methods

### Participants

**Figure 1:**
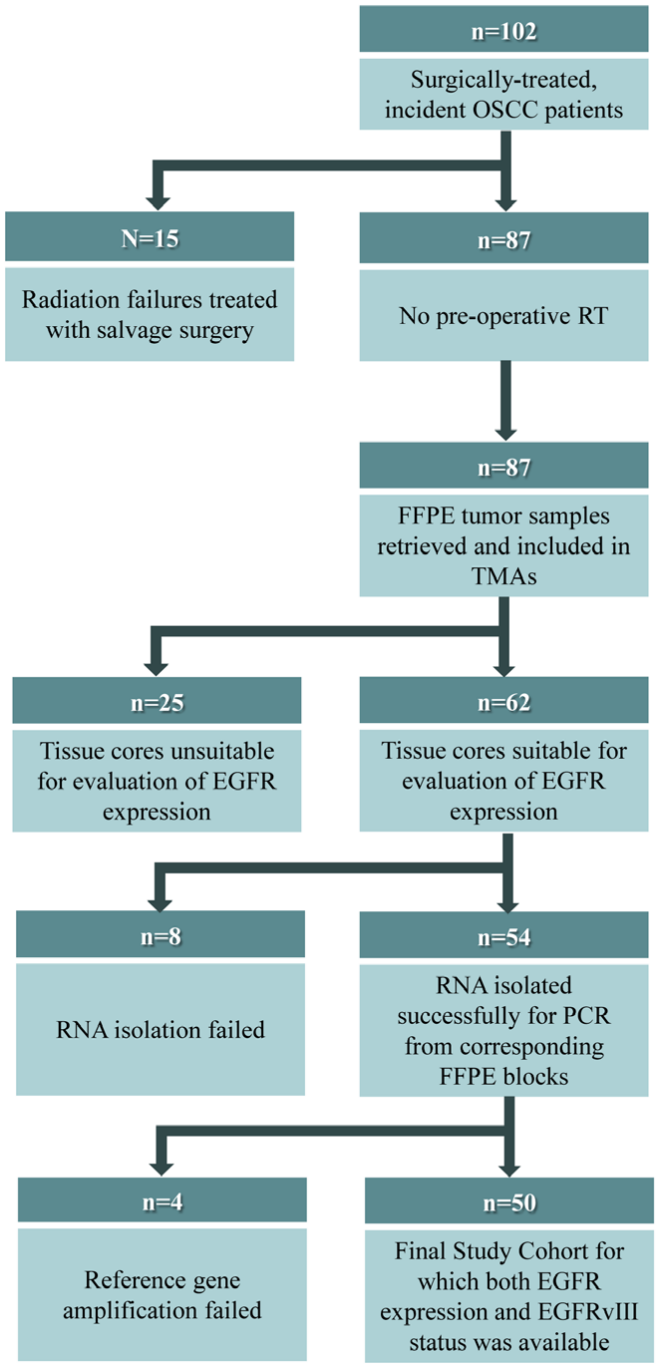
CONSORT Diagram outlining patient selection criteria and the different stages at which specific analyses were undertaken for the OSCC study cohort.

The study cohort consisted of 102 patients with OSCC who were treated with primary surgery (resection and neck dissection) between 1998 and 2005 at the Foothills Medical Centre, Calgary, Alberta, Canada. The inclusion criteria for patients were the presentation of OSCC with no prior history or treatment for head and neck cancer. Of the 102 patients with OSCC, 87 satisfied these inclusion criteria. Of these 87 patients who were included in tissue microarrays (TMAs), 62 had tissue cores suitable for quantitative immunohistochemistry. For *EGFRvIII* analysis, RNA extraction from archived FFPE tumor samples was successful in 54 out of 62 patients. Reference gene amplification failed in 4 patients. These details are outlined in the CONSORT diagram (Figure 1). The clinico-pathological characteristics of the patients included in this study are represented in Table 1. In order to address possible sample selection bias due to tumor heterogeneity we randomly selected 22 additional FFPE tumor blocks including the *EGFRvIII*-positive patient, for duplicate *EGFRvIII* analysis (Table S3). Therefore, a total of 72 tumor blocks were tested for *EGFRvIII* expression. Patient demographics and clinical outcomes were collected by a combination of comprehensive chart review and data retrieved from the Alberta Cancer Registry.

**Table 1:** Clinicopathological characteristics of OSCC patients evaluated for EGFR protein expression and *EGFRvIII* frequency.

	n = 54
**Sex (n, %)**	
**Male**	38 (70%)
**Female**	16 (30%)
**Age (mean, sd)**	61 (12.3)
**Smoking History (n, %)**	
**Never**	12 (22%)
**Past**	12 (22%)
**Current**	30 (56%)
**Alcohol History (n,%)**	
**Never**	6 (11%)
**Moderate**	19 (35%)
**Heavy**	15 (28%)
**Former Heavy**	10 (19%)
**Not Stated**	4 (7%)
**Pathologic T Stage (n, %)**	
**pT1**	8 (15%)
**pT2**	19 (35%)
**pT3**	12 (22%)
**pT4**	15 (28%)
**Pathologic N Stage (n, %)**	
**pN0**	28 (52%)
**pN1**	7 (13%)
**pN2**	19 (35%)
**Differentiation (n, %)**	
**Well**	10 (19%)
**Moderate**	31 (57%)
**Poor**	6 (11%)
**Not Stated**	7 (13%)

### Description of Procedures

**RNA isolation and cDNA synthesis:** 2–3 slices of 10 µm thickness were sectioned from FFPE tumor blocks were and placed in 1.5 ml centrifuge tubes. Tissues were de-paraffinized by treatment with xylene and ethanol. Total RNA was isolated using RecoverAll™ Total Nucleic Acid Isolation kit (Ambion, Austin, TX) according to manufacturer's protocol. The U87MG cell line was purchased from American Type Culture Collection (ATCC, Manassas, VA); the U87MGvIII cell line was a gift from Dr. Paul S. Mischel. Total RNA was isolated from both cell lines using Trizol (Invitrogen, Carlsbad, CA) according to manufacturer's protocol. For all samples RNA concentration and purity was determined using a Nano-Drop 2000C spectrophotometer (Thermo Scientific, Wilmington, DE). A260/A280 nm was >1.9 for most samples. For each sample, 1 µg of RNA was used as template in the reverse transcription reaction using qScript™ cDNA SuperMix (Quanta BioSciences, Gaithersburg, MD) in a 20 µl volume according to manufacturer's protocol.

**Real-time PCR:** Real-time PCR was performed on the ABI 7500 Real Time system using TaqMan® probe-based chemistry (Applied Biosystems, Carlsbad, CA). An *EGFRvIII*-specific gene expression assay was designed using the File Builder 3.1 software (Applied Biosystems, Carlsbad, CA). The following primer/probe set was used: forward primer 5′ TCTGCCCGGCGAGTC 3′, reverse primer 5′ GCCGTGATCTGTCACCACATAATT 3′, FAM-labelled probe 5′ TTTCTTTTCCTCCAGAGCCC 3′. Briefly, 2 µl of cDNA product was used as template in a 50 µl PCR reaction containing 25 µl TaqMan® Universal PCR Master Mix (2×), 2.5 µl *EGFRvIII* primer/probe mix and 20.5 µl nuclease-free water. The following amplification protocol was performed: 50°C for 2 minutes, 95°C for 10 minutes; followed by 40 cycles of 95°C for 15 seconds each and finally 60°C for 1 minute. Fluorescent signals were collected during the 60°C/1 minute step. Baseline and threshold cycle number (Ct) were determined manually. *EGFRvIII* PCR efficiency and linear dynamic range was assessed by using the following dilution series: 100 ng, 10 ng, 1 ng, 0.1 ng and 0.01 ng of U87MGvIII cDNA in triplicate. Both *Beta-2-microglobulin (B2M)* and *GAPDH* served as reference genes.

The glioblastoma cell line U87MGvIII over-expresses EGFRvIII and was used as a positive control; the parental cell line, U87MG expresses only wild-type EGFR and served as a negative control. Water was used as a no template control for the PCR reaction. *EGFRvIII* positive samples were retested to confirm the presence of *EGFRvIII* and the mean Ct number was used for data analysis. Relative expression of *EGFRvIII* was determined by delta Ct method which was calculated by subtracting the average Ct reference value from that of *EGFRvIII*.

**Conventional RT-PCR and Sequencing:** cDNA was sequenced to verify *EGFRvIII* mutational status and validate the novel *EGFRvIII* rt RT-PCT assay. A nested PCR strategy was employed to maximize yield from FFPE tumor samples. Briefly, 2 µl of cDNA were amplified in the first round PCR in a total volume of 50 µl containing 1× High Fidelity PCR Buffer, 0.2 mM dNTPs, 2 mM MgSO_4_, 20 pmol of each primer and 1 unit of Platinum Taq High Fidelity polymerase (Invitrogen, Carlsbad, CA). Primers are located in exon 1 and exon 10 as described previously [29]. PCR cycling conditions were: 94°C for 2 minutes followed by 43 cycles of 94°C/30 s, 55°C/30 s, 72°C/1 min and a final extension step of 72°C for 10 minutes. 1 µl of first round PCR reaction was used as template in a second round amplification using previously reported primers and conditions with the exception that amplification was carried out for 30 cycles instead of 42 [30]. Nested PCR products were purified and resuspended in 30 µl of elution buffer using the QIAquick PCR Purification kit (Qiagen, Valencia, CA). Bi-directional sequencing was performed using BigDye® Terminator v3.1 Sequencing kit (ABI) using the following primers: 5′TTCGGGGAGCACCGATGCGAC3′ (EGFR exon 1) and 5′ACCAATACCTATTCCGTTACAC3′ (EGFR exon 9). Unincorporated dye was removed using the DyeEx® 2.0 Spin Kit (Qiagen, Valencia, CA) and capillary electrophoresis was performed on the 3130 Genetic Analyzer (ABI).

**Tissue Microarray (TMA) Construction:** Formalin-fixed paraffin–embedded (FFPE) tumor samples from patients included in the study were retrieved for tissue microarray (TMA) construction. Hematoxylin and eosin stained slides were reviewed by the study pathologist (A.M) to confirm diagnosis and those deemed to be of sufficient quality were selected and marked for sampling and inclusion into TMAs. TMAs were assembled from triplicate 0.6 mm cores of FFPE primary tumor samples using a Beecher Manual Tissue Microarrayer (Beecher Instruments Inc. Sun Prairie, WI). Five samples of normal oral cavity squamous epithelium were also included in the TMAs as reference samples for normal biomarker expression levels.

**Quantitative Fluorescent Immunohistochemistry:** We used the HistoRx AQUA® platform and fluorescent immunohistochemistry [31] to quantify the expression of EGFR protein in the tumor (epithelial) compartment of each TMA core. TMA sections (4 µm) were deparaffinized in xylene, rinsed in ethanol, and rehydrated. Heat induced epitope retrieval was performed by heating slides to 121°C in a citrate-based buffer (pH 6.0) Target Retrieval Solution (DAKO) for 6 minutes in a decloaking chamber (Biocare Medical). TMA slides were stained for rabbit monoclonal anti-EGFR antibody (1∶1000 dilution, Epitomics) for 60 minutes. Rabbit Envision+ kit (DAKO) was used in conjunction with tyramide-Cy5 (Perkin-Elmer) to visualize EGFR expression. The epithelial (tumor) compartment was identified by staining with a guinea-pig anti-pan-cytokeratin (PCK) antibody (ACRIS) for 60 minutes and an Alexa488 conjugated anti-guinea pig secondary antibody for 60 minutes. Slides were scanned by HistoRx PM-2000™ and analyzed by AQUA® software (version 2.2.1.7): the tumor compartment was defined as the PCK-positive area for each TMA core. AQUA® software calculated the EGFR expression from the average exposure time-adjusted EGFR pixel intensity density from each compartment. The average score from triplicate cores was reported for the 50 patients (51 tissue blocks) for whom both AQUA scores and real-time PCR data for *EGFRvIII* expression were available.

**Ethics:** This study was approved by the Conjoint Health Research Ethics Board of the University of Calgary. Written consent for access to personal health information was waived under Section 50 of the Alberta Health Information Act on grounds that it was not feasible.

## Results

### EGFRvIII Detection in Cell lines

**Figure 2:**
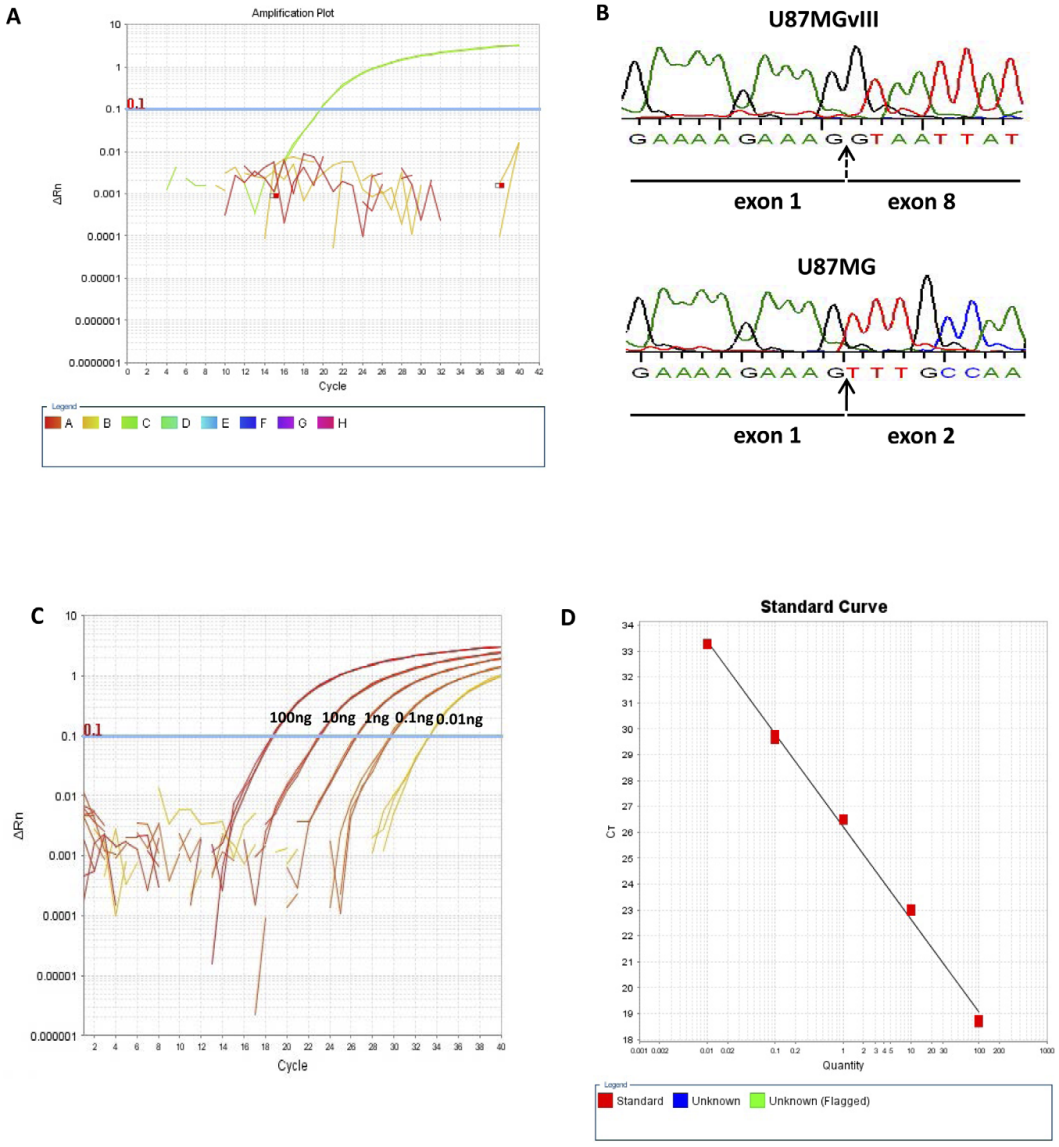
Specific detection of *EGFRvIII* mRNA by real-time RT-PCR. A. Real-time amplification plot of *EGFRvIII* transcript as detected in the U87MGvIII cell-line. U87MGvIII cells were used as a positive control, U87MG cells were used as negative control: water was used for no template control. B. Direct sequencing of U87MG and U87MGvIII cDNA confirms the presence of *EGFRvIII* transcript in U87MGvIII cells only. The broken arrow indicates the *EGFRvIII*-specific exon 1 and exon 8 junction. C. Linear dynamic range of the *EGFRvIII* real-time RT-PCR assay (100, 10, 1, 0.1 and 0.01 ng cDNA). D. *EGFRvIII* real-time RT-PCR assay amplification efficiency.

We detected the presence of *EGFRvIII* in U87MGvIII cells and confirmed the absence of *EGFRvIII* in U87MG cells using our novel real-time RT-PCR assay. These results support the analytical specificity of our real-time RT-PCR *EGFRvIII* detection method since U87MGvIII cells express both wild-type *EGFR* and *EGFRvIII*, and the parental cell line U87MG only expresses wild-type EGFR. Strong amplification curves were generated using U87MGvIII cDNA as template in real-time PCR reactions (Figure 2A). In contrast, no amplification was evident in reactions using the U87MG cDNA template, indicating that our assay is specific for the detection of the *EGFRvIII* mutation and does not amplify wild-type *EGFR*.

We also confirmed the expression of *EGFRvIII* transcript by direct sequencing of cDNA from both U87MG and U87MGvIII. *EGFRvIII* was only detected in the U87MGvIII cell line by direct sequencing, thus confirming the results obtained using our novel real-time RT-PCR assay (Figure 2B). We assessed the sensitivity and PCR efficiency of our assay by titrating a broad range of cDNA template concentrations. *EGFRvIII* amplification was achieved with template amounts as low as 10 pg. The calibration curves (Figure 2C and 2D) demonstrate the robust linear dynamic range and high PCR efficiency (90%) of our novel assay; these are essential attributes for sensitive and precise real-time PCR assays.

### EGFRvIII Detection in Glioblastoma FFPE Tissue

The frequency of *EGFRvIII* mutations in glioblastoma (∼30%) is well established in the literature [15]. Consequently, we profiled *EGFRvIII* expression in tumors from a cohort of 26 glioblastoma patients to test the *EGFRvIII* detection performance of our real-time RT-PCR assay in FFPE tumor samples. For comparison, we also attempted *EGFRvIII* detection using conventional RT-PCR and direct sequencing, in the same samples. Conventional end-point RT-PCR was successful in only 7/26 samples (4 positive and 3 negative for *EGFRvIII*), while target amplification failed in the remaining 19 cases. Direct sequencing gave slightly better results; 10/26 samples produced adequate quality electropherographic reads (5 positive and 5 negative for *EGFRvIII*) but electropherograms were inadequate for samples from the remaining 16 tumors.

When performing our real-time RT-PCR assay on the glioblastoma samples, we used *B2M* as the reference gene. *B2M* was amplified successfully in all samples (26/26), while *EGFRvIII* was amplified in 10/26 samples. The detection of *EGFRvIII* by conventional RT-PCR and our novel real-time RT-PCR analysis were fully concordant; all samples that were *EGFRvIII*-positive by conventional RT-PCR were also *EGFRvIII*-positive according to our real-time RT-PCR analysis. Additionally, of the three tumor samples that were *EGFRvIII*-negative (expressing only *wtEGFR*) according to RT-PCR, two were re-classified as *EGFRvIII*-positive by our real-time RT-PCR assay; these tumor samples had high *EGFRvIII* Ct values and very high ΔCt values (Table S1).

The detection of *EGFRvIII* by our novel real-time RT-PCR method was fully concordant with the detection of *EGFRvIII* by direct sequencing in glioblastoma FFPE samples; all samples that tested positive for *EGFRvIII* by direct sequencing were also *EGFRvIII*-positive according to our novel real-time RT-PCR assay. Of the five samples that were *EGFRvIII*-negative by direct sequencing, two were re-classified as *EGFRvIII*-positive by our novel real-time RT-PCR method. These two samples only amplified a wild-type EGFR product by conventional RT-PCR but had high *EGFRvIII* Ct and ΔCt values when analyzed by our novel real-time RT-PCR. These results demonstrate the low sensitivity and inherent limitations of conventional PCR and direct sequencing methods when applied to FFPE tissue samples. Compromised RNA quality and fragmentation limits the size of amplicons that can be generated from FFPE tissue, thus rendering them unsuitable for conventional PCR and direct sequencing applications. Another possibility might be the low abundance of the *EGFRvIII* transcript in these samples that may be attributed to the tumor heterogeneity of glioblastomas.

### EGFRvIII Detection in OSCC FFPE Tissue

**Figure 3:**
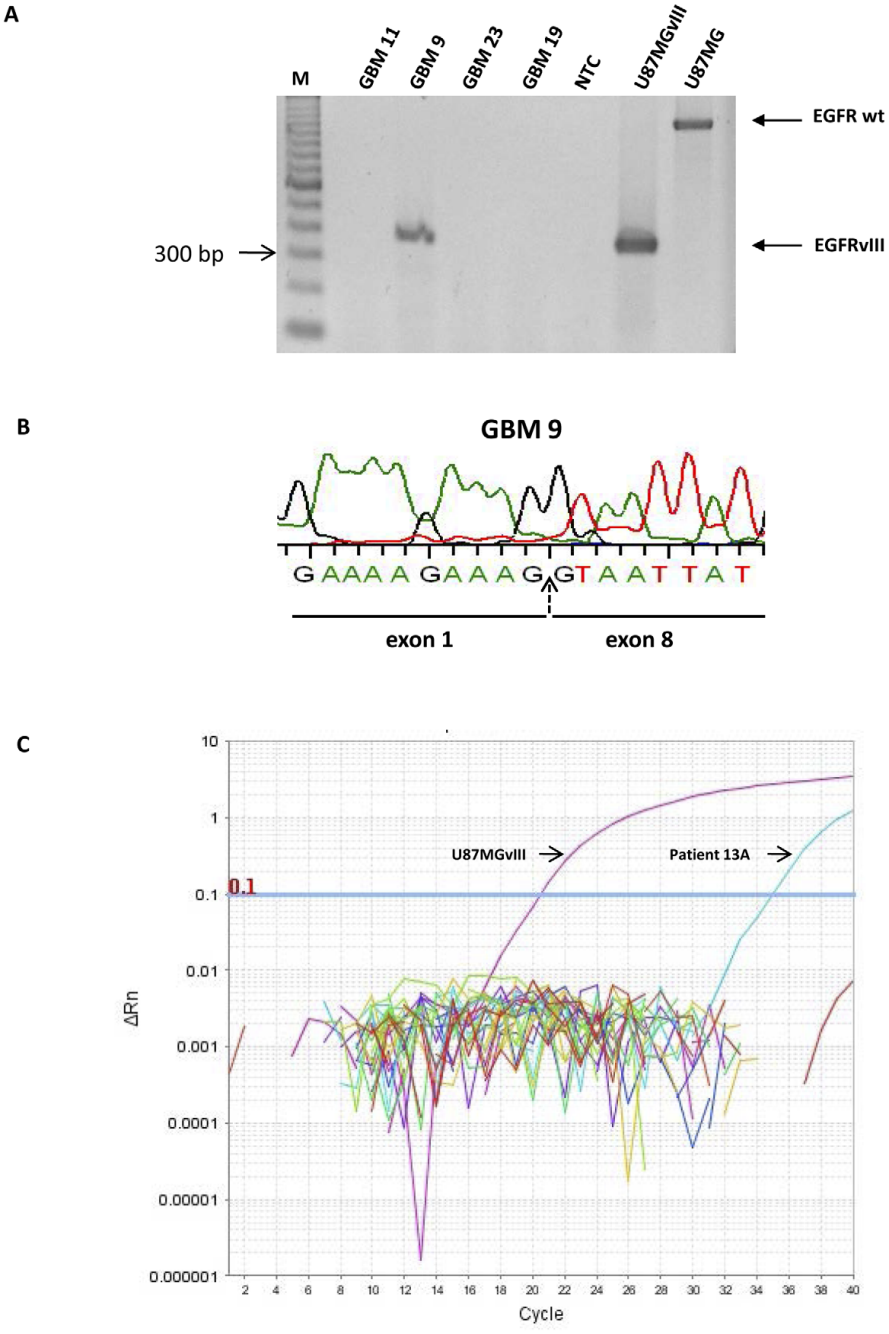
Conventional RT-PCR and direct cDNA sequencing confirm the presence of *EGFRvIII* in glioblastoma FFPE tissue. A. Conventional RT-PCR failed for GBM 11, GBM 23 and GBM 19. GBM 9 is positive for *EGFRvIII*. EGFRvIII positive control: U87MGvIII (384 bp), negative control: U87MG (1184 bp) and no template control (NTC): water. B. Sequencing of GBM 9 cDNA confirms the presence of *EGFRvIII*. C. Real-time amplification plot showing EGFRvIII-positive OSCC patient (13A) and the positive control (U87MGvIII).

We evaluated tumor samples from 54 OSCC patients for the presence of *EGFRvIII* using our novel real-time RT-PCR assay. Four samples were excluded from the analysis either due to failed amplification of the reference genes (*B2M and GAPDH*) or having Ct values greater than 38 for one or both of these reference genes. All other samples successfully amplified both reference genes. Our real time PCR assay revealed that only one patient (1/50) was positive for *EGFRvIII* mRNA expression (Figure 3C and Table S2). Retesting of this sample confirmed *EGFRvIII* positivity. In addition, both direct cDNA sequencing and conventional RT-PCR were performed on this sample, however both methods failed outright. This failure may be attributed to formalin fixation-induced degradation and modification of DNA/RNA and further highlights the limitations of conventional methods when using FFPE tissue.

**Figure 4:**
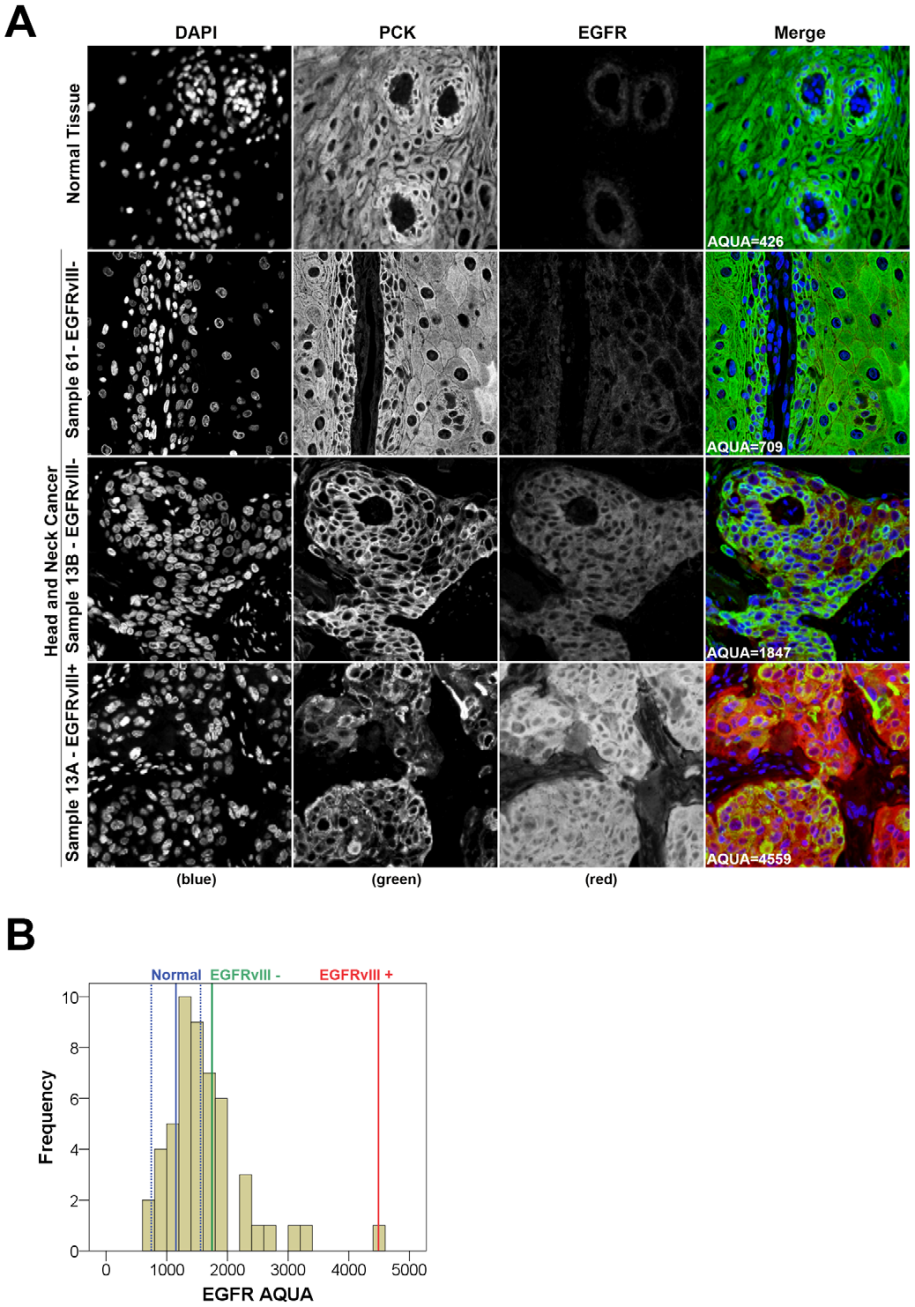
Immunofluorescent staining and quantitative analysis of EGFR protein expression in OSCC samples using the HistoRx AQUA® platform. A. Representative examples of normal oral cavity squamous epithelium in the top panel, a low expressing tumor from an *EGFRvIII*-negative patient in the top-mid panel, and the EGFRvIII negative and EGFRvIIIitive samples from patient 13 in the bottom two panels. DAPI (blue) = nuclei, pan-cytokeratin (green) = epithelial/tumor cells, and EGFR (red). B. Histogram distribution of the EGFR AQUA scores for the entire OSCC patient cohort. The red and green lines indicate the EGFR scores for patient 13 who had both an *EGFRvIII*-positive (red, Sample ID 13A) and *EGFRvIII*-negative (green, Sample ID 13B) tumor sample. The solid blue line indicates the median EGFR AQUA score for normal oral cavity squamous epithelium, surrounded by the hashed blue lines representing one standard deviation above and below the median.

We also measured total EGFR protein levels for all samples by quantitative fluorescent immunohistochemistry using the HistoRx AQUA® platform [31] (Figure 4A). EGFR AQUA scores, representing the concentration of EGFR protein, showed a range of expression from 426 to 1696 in normal oral cavity squamous epithelium with a median score of 1151 and a standard deviation of ±406 (Figure 4B, blue lines). We used the median EGFR AQUA score plus one standard deviation (AQUA = 1557) as our definition for EGFR over-expression. Using this definition, we found that tumors from 22 of 50 patients over-expressed EGFR. Comparison of EGFR AQUA scores with *EGFRvIII* expression showed that the tumor sample with the highest EGFR AQUA score was the *EGFRvIII*-positive case identified by our real-time RT-PCR assay (Figure 4A, bottom panel; Figure 4B red line; Table S2). In order to address tumor heterogeneity, an additional FFPE tumour block was randomly selected from 22 patients in the cohort and was tested for *EGFRvIII* expression (see methods section for details; Table S3). The additional samples included a tumor block from the patient that had tested positive for *EGFRvIII*. *EGFRvIII* transcript was not present in any of these additional tumor samples (Table S3). Furthermore, AQUAnalysis® of the *EGFRvIII*-negative sample obtained from the single *EGFRvIII-*positive patient showed this sample to have significantly lower wild-type EGFR protein expression (Table S2). Taken together, these results suggest that *EGFRvIII* expression in OSCC is a rare event (2%) and most likely to be present in tumors which express very high levels of EGFR protein.

## Discussion

We have developed a highly sensitive and specific real-time RT-PCR assay for *EGFRvIII* detection. We validated our assay in a cohort of glioblastoma patients and compared its efficiency to conventional PCR and direct sequencing. Furthermore, in light of the conflicting reports regarding the frequency of *EGFRvIII* in HNSCC [21], [22], [23], [25], we investigated the frequency of *EGFRvIII* in OSCC, the most prevalent form of HNSCC, using our novel technique. Despite the highly sensitive nature of our assay, we only detected a single *EGFRvIII*-positive patient in our OSCC cohort of 50 patients. This discrepancy between our novel real-time RT-PCR assay results and the reported frequency of *EGFRvIII* in HNSCC may be due to differing *EGFRvIII* mutation frequencies in specific HNSCC subsites or selection bias inherent in early phase clinical trials. Patient eligibility for such trials was based on recurrent or metastatic disease status or failure of first-line therapy [23]. Since *EGFRvIII*-positive tumors are expected to be less responsive to conventional therapies than *EGFRvIII*-negative tumors, it would be expected that *EGFRvIII*-positive tumors would be over-represented in these cohorts. The cohort in the current study included patients with no prior treatment for HNSCC and may more accurately reflect the frequency of *EGFRvIII* in this disease site. The low *EGFRvIII* frequency reported in another unselected HNSCC cohort [21] supports this assertion.

Since EGFR over-expression and amplification of the *EGFR* locus has been linked to the presence of EGFRvIII [9], we used quantitative immunohistochemistry and AQUA® technology to measure EGFR expression in TMAs constructed from our patient tumor samples. The single OSCC patient that tested positive for *EGFRvIII* also exhibited the highest expression of EGFR protein. This increased staining may be attributed to the presence of both wild-type and mutant forms of EGFR, since our EGFR antibody detects both wild-type EGFR and EGFRvIII. Also, the absence of *EGFRvIII* in an additional tumor sample from the same patient, and expressing significantly lower levels of EGFR, corroborates reports suggesting that EGFR over-expression is linked to the presence of *EGFRvIII*. The apparent mosaic pattern of *EGFRvIII* expression in the *EGFRvIII*-positive patient also confirms the heterogeneity of OSCC similar to malignant glioblastomas, where the proportion of *EGFRvIII*-expressing cells has been shown to range from 37% to 86% in *EGFRvIII-*positive tumor samples [32].

The cancer-specific *EGFRvIII* deletion mutant is the focus of investigation in a variety of cancers. *In vitro* and *in vivo* studies attest to the increased tumorogenicity of *EGFRvIII* expressing cells [22], thus making it a prime target for novel therapies. However, the implementation of these therapies is contingent on the accurate and sensitive detection of *EGFRvIII* in tumor samples. A variety of methods are available for the detection of *EGFRvIII* in clinical specimens. Fresh frozen tissue samples are amenable to conventional RT-PCR and direct sequencing-based detection methods but these techniques are unreliable in FFPE samples. RNA isolated from paraffin-embedded tissue is often highly degraded and compromised by cross-linking and modifications introduced during the fixation process. This can prove challenging for downstream applications such as PCR where high nucleic acid integrity is essential. Immunohistochemical detection of EGFRvIII protein can achieve high sensitivity and specificity, attributed to the presence of a unique glycine residue in *EGFRvIII*. However, the widespread clinical use of EGRFvIII immunohistochemistry is restricted by patents and availability of antibodies. Several real-time PCR-based techniques have also been described in the literature and their utility in FFPE tumor samples has been successfully demonstrated. A majority of these techniques use dye-based chemistries and are less specific than probe-based assays, rendering them more prone to the detection of false positives. Also, the sensitivity of some of these real-time PCR-based methods may be inadequate for detecting trace amounts of *EGFRvIII* mRNA that might be present in early stage tumor samples. Our novel rt RT-PCT assay is less prone to the detection of false positives due to its specificity for EGFRvIII. It is optimized for the detection of *EGFRvIII* in FFPE tumor samples because of its sensitivity towards small amplicon sizes and its inability to detect the presence of wild-type EGFR.

This is the first report describing the frequency of *EGFRvIII* specifically in OSCC. Despite advances in therapy, long-term survival in OSCC patients remains poor. The overall survival rate, worldwide, from oral cancer is generally less than 50% and has remained unchanged for more than three decades [33], [34]. Recently, there has been a concerted effort towards the development of EGFR-targeted therapies in OSCC. Also, several groups have advocated the use of EGFRvIII-specific therapies in HNSCC patients. Our results suggest that EGFRvIII-specific therapies may not be ideally suited as first-line treatment in OSCC due to the low occurrence of *EGFRvIII* at this subsite. However, EGFRvIII targeting might be a valuable addition to therapeutic regimens in recurrent/metastatic OSCC where *EGFRvIII* might be over-represented. Since tumors expressing *EGFRvIII* are refractory to EGFR-targeted therapy [22], this may explain the poor success of EGFR targeting in clinical trials involving HNSCC patients [35], [36], [37]. Therefore, testing for *EGFRvIII* frequency could provide greater benefits in patients with advanced disease and treatment failures.

Our AQUAnalysis® suggests that EGFR is over-expressed in 44% of OSCC. This is in contrast with reports where EGFR over-expression has been described in ∼100% of OSCC [38]. The discrepancy in the reported frequency of EGFR protein over-expression in OSCC could be attributed to our more stringent definition of EGFR over-expression; we used quantitative fluorescent immunohistochemistry for the assessment of EGFR expression in normal and malignant tissue and defined EGFR over-expression relative to EGFR expression in normal tissue. Additionally, the automated, observer-independent nature of AQUA® yields highly reproducible protein expression data on a continuous scale, providing better resolution between patients than conventional semi-quantitative IHC.

We conclude that highly specific and sensitive methods, such as the real-time RT-PCR assay and quantitative fluorescent immunohistochemistry described in this research, are essential for accurate assessment of EGFR expression and EGFR mutations, and will facilitate the selection of optimal tailored therapies for OSCC patients. Also, improved screening methods for EGFR and other cancer-specific abnormalities, should be incorporated into routine clinical diagnostic testing to pave the way for early diagnosis and improved survival in OSCC. In keeping with this goal, several prominent cancer centers are already using the routine analysis of biopsies to identify gene mutations and triage cancer patients for targeted therapies [39].

### Limitations

We studied a relatively small retrospective cohort of OSCC patients. Translation of our findings into changes in clinical practice would require confirmation in a prospective clinical setting.

## Supporting Information

Table S1 **Validation of novel real-time RT-PCR assay in glioblastoma patient samples.**
(XLS)

Table S2 **Results for** ***EGFRvIII*** **real-time RT-PCR assay and EGFR AQUAnalysis® performed on OSCC patient.**
(XLS)

Table S3 **Results for** ***EGFRvIII*** **real-time RT-PCR assay performed on the additional blocks obtained from 22 of the 50 OSCC patients included in the study.**
(XLS)

## References

[1] Harris TJ, McCormick F (2010). The molecular pathology of cancer.. Nature reviews Clinical oncology.

[2] Mitsudomi T, Yatabe Y (2010). Epidermal growth factor receptor in relation to tumor development: EGFR gene and cancer.. The FEBS journal.

[3] Lai SY, Johnson FM (2010). Defining the role of the JAK-STAT pathway in head and neck and thoracic malignancies: implications for future therapeutic approaches.. Drug resistance updates: reviews and commentaries in antimicrobial and anticancer chemotherapy.

[4] Zhang Z, Stiegler AL, Boggon TJ, Kobayashi S, Halmos B (2010). EGFR-mutated lung cancer: a paradigm of molecular oncology.. Oncotarget.

[5] Moscatello DK, Holgado-Madruga M, Godwin AK, Ramirez G, Gunn G (1995). Frequent expression of a mutant epidermal growth factor receptor in multiple human tumors.. Cancer research.

[6] Wikstrand CJ, Hale LP, Batra SK, Hill ML, Humphrey PA (1995). Monoclonal antibodies against EGFRvIII are tumor specific and react with breast and lung carcinomas and malignant gliomas.. Cancer research.

[7] Batra SK, Castelino-Prabhu S, Wikstrand CJ, Zhu X, Humphrey PA (1995). Epidermal growth factor ligand-independent, unregulated, cell-transforming potential of a naturally occurring human mutant EGFRvIII gene.. Cell growth & differentiation: the molecular biology journal of the American Association for Cancer Research.

[8] Biernat W, Huang H, Yokoo H, Kleihues P, Ohgaki H (2004). Predominant expression of mutant EGFR (EGFRvIII) is rare in primary glioblastomas.. Brain pathology.

[9] Sasaki H, Kawano O, Endo K, Yukiue H, Yano M (2007). EGFRvIII mutation in lung cancer correlates with increased EGFR copy number.. Oncology reports.

[10] Lammering G, Hewit TH, Holmes M, Valerie K, Hawkins W (2004). Inhibition of the type III epidermal growth factor receptor variant mutant receptor by dominant-negative EGFR-CD533 enhances malignant glioma cell radiosensitivity.. Clinical cancer research: an official journal of the American Association for Cancer Research.

[11] Montgomery RB, Guzman J, O'Rourke DM, Stahl WL (2000). Expression of oncogenic epidermal growth factor receptor family kinases induces paclitaxel resistance and alters beta-tubulin isotype expression.. The Journal of biological chemistry.

[12] Yamazaki H, Ohba Y, Tamaoki N, Shibuya M (1990). A deletion mutation within the ligand binding domain is responsible for activation of epidermal growth factor receptor gene in human brain tumors.. Japanese journal of cancer research: Gann.

[13] Ekstrand AJ, Sugawa N, James CD, Collins VP (1992). Amplified and rearranged epidermal growth factor receptor genes in human glioblastomas reveal deletions of sequences encoding portions of the N- and/or C-terminal tails.. Proceedings of the National Academy of Sciences of the United States of America.

[14] Wong AJ, Ruppert JM, Bigner SH, Grzeschik CH, Humphrey PA (1992). Structural alterations of the epidermal growth factor receptor gene in human gliomas.. Proceedings of the National Academy of Sciences of the United States of America.

[15] Heimberger AB, Hlatky R, Suki D, Yang D, Weinberg J (2005). Prognostic effect of epidermal growth factor receptor and EGFRvIII in glioblastoma multiforme patients.. Clinical cancer research: an official journal of the American Association for Cancer Research.

[16] Garcia de Palazzo IE, Adams GP, Sundareshan P, Wong AJ, Testa JR (1993). Expression of mutated epidermal growth factor receptor by non-small cell lung carcinomas.. Cancer research.

[17] Ji H, Zhao X, Yuza Y, Shimamura T, Li D (2006). Epidermal growth factor receptor variant III mutations in lung tumorigenesis and sensitivity to tyrosine kinase inhibitors.. Proceedings of the National Academy of Sciences of the United States of America.

[18] Olapade-Olaopa EO, Moscatello DK, MacKay EH, Horsburgh T, Sandhu DP (2000). Evidence for the differential expression of a variant EGF receptor protein in human prostate cancer.. British journal of cancer.

[19] Ozanne B, Richards CS, Hendler F, Burns D, Gusterson B (1986). Over-expression of the EGF receptor is a hallmark of squamous cell carcinomas.. The Journal of pathology.

[20] Grandis JR, Tweardy DJ (1993). TGF-alpha and EGFR in head and neck cancer.. Journal of cellular biochemistry Supplement.

[21] Hama T, Yuza Y, Saito Y, Ou J, Kondo S (2009). Prognostic significance of epidermal growth factor receptor phosphorylation and mutation in head and neck squamous cell carcinoma.. The oncologist.

[22] Sok JC, Coppelli FM, Thomas SM, Lango MN, Xi S (2006). Mutant epidermal growth factor receptor (EGFRvIII) contributes to head and neck cancer growth and resistance to EGFR targeting.. Clinical cancer research: an official journal of the American Association for Cancer Research.

[23] Chau NG, Perez-Ordonez B, Zhang K, Pham NA, Ho J (2011). The association between EGFR variant III, HPV, p16, c-MET, EGFR gene copy number and response to EGFR inhibitors in patients with recurrent or metastatic squamous cell carcinoma of the head and neck.. Head & neck oncology.

[24] Leemans CR, Braakhuis BJ, Brakenhoff RH (2011). The molecular biology of head and neck cancer.. Nature reviews Cancer.

[25] Yang B, Chen J, Zhang X, Cao J (2009). Expression of Epidermal Growth Factor Receptor variant III in laryngeal carcinoma tissues.. Auris, nasus, larynx.

[26] Takes RP, Baatenburg de Jong RJ, Schuuring E, Litvinov SV, Hermans J (1998). Differences in expression of oncogenes and tumor suppressor genes in different sites of head and neck squamous cell.. Anticancer research.

[27] Bernier J, Bentzen SM, Vermorken JB (2009). Molecular therapy in head and neck oncology.. Nature reviews Clinical oncology.

[28] Yoshimoto K, Dang J, Zhu S, Nathanson D, Huang T (2008). Development of a real-time RT-PCR assay for detecting EGFRvIII in glioblastoma samples.. Clinical cancer research: an official journal of the American Association for Cancer Research.

[29] Frederick L, Eley G, Wang XY, James CD (2000). Analysis of genomic rearrangements associated with EGRFvIII expression suggests involvement of Alu repeat elements.. Neuro-oncology.

[30] Mellinghoff IK, Wang MY, Vivanco I, Haas-Kogan DA, Zhu S (2005). Molecular determinants of the response of glioblastomas to EGFR kinase inhibitors.. The New England journal of medicine.

[31] Camp RL, Chung GG, Rimm DL (2002). Automated subcellular localization and quantification of protein expression in tissue microarrays.. Nature medicine.

[32] Wikstrand CJ, McLendon RE, Friedman AH, Bigner DD (1997). Cell surface localization and density of the tumor-associated variant of the epidermal growth factor receptor, EGFRvIII.. Cancer research.

[33] Vokes EE, Weichselbaum RR, Lippman SM, Hong WK (1993). Head and neck cancer.. The New England journal of medicine.

[34] Farah CS, McCullough MJ (2008). Oral cancer awareness for the general practitioner: new approaches to patient care.. Australian dental journal.

[35] Soulieres D, Senzer NN, Vokes EE, Hidalgo M, Agarwala SS (2004). Multicenter phase II study of erlotinib, an oral epidermal growth factor receptor tyrosine kinase inhibitor, in patients with recurrent or metastatic squamous cell cancer of the head and neck.. Journal of clinical oncology: official journal of the American Society of Clinical Oncology.

[36] Siu LL, Soulieres D, Chen EX, Pond GR, Chin SF (2007). Phase I/II trial of erlotinib and cisplatin in patients with recurrent or metastatic squamous cell carcinoma of the head and neck: a Princess Margaret Hospital phase II consortium and National Cancer Institute of Canada Clinical Trials Group Study.. Journal of clinical oncology: official journal of the American Society of Clinical Oncology.

[37] Stewart JS, Cohen EE, Licitra L, Van Herpen CM, Khorprasert C (2009). Phase III study of gefitinib compared with intravenous methotrexate for recurrent squamous cell carcinoma of the head and neck [corrected].. Journal of clinical oncology: official journal of the American Society of Clinical Oncology.

[38] Monteiro LS, Diniz-Freitas M, Garcia-Caballero T, Forteza J, Fraga M (2010). EGFR and Ki-67 expression in oral squamous cell carcinoma using tissue microarray technology.. Journal of oral pathology & medicine: official publication of the International Association of Oral Pathologists and the American Academy of Oral Pathology.

[39] Pao W, Kris MG, Iafrate AJ, Ladanyi M, Janne PA (2009). Integration of molecular profiling into the lung cancer clinic.. Clinical cancer research: an official journal of the American Association for Cancer Research.

